# MYRF: A New Regulator of Cardiac and Early Gonadal Development—Insights from Single Cell RNA Sequencing Analysis

**DOI:** 10.3390/jcm11164858

**Published:** 2022-08-18

**Authors:** Verónica Calonga-Solís, Helena Fabbri-Scallet, Fabian Ott, Mostafa Al-Sharkawi, Axel Künstner, Lutz Wünsch, Olaf Hiort, Hauke Busch, Ralf Werner

**Affiliations:** 1 Division of Paediatric Endocrinology and Diabetes, Department of Paediatric and Adolescent Medicine, University of Lübeck, 23562 Lübeck, Germany; 2 Medical Systems Biology Division, Lübeck Institute of Experimental Dermatology, University of Lübeck, 23562 Lübeck, Germany; 3 Center for Molecular Biology and Genetic Engineering—CBMEG, State University of Campinas, Campinas 13083-875, Brazil; 4 Biochemical Genetics Department, Human Genetics and Genome Research Institute, National Research Centre, Dokki, Cairo 12622, Egypt; 5 Department of Pediatric Surgery, University of Lübeck, 23562 Lübeck, Germany; 6 Institute of Molecular Medicine, University of Lübeck, 23562 Lübeck, Germany

**Keywords:** *MYRF*, *CITED2*, scimitar syndrome, disorders of sex development, gonadal development, cardiac and urogenital syndrome

## Abstract

De novo variants in the myelin regulatory factor (MYRF), a transcription factor involved in the differentiation of oligodendrocytes, have been linked recently to the cardiac and urogenital syndrome, while familiar variants are associated with nanophthalmos. Here, we report for the first time on a patient with a de novo stop-gain variant in *MYRF* (p.Q838*) associated with Scimitar syndrome, 46,XY partial gonadal dysgenesis (GD) and severe hyperopia. Since variants in *MYRF* have been described in both 46,XX and 46,XY GD, we assumed a role of *MYRF* in the early development of the bipotential gonad. We used publicly available single cell sequencing data of human testis and ovary from different developmental stages and analysed them for *MYRF* expression. We identified *MYRF* expression in the subset of coelomic epithelial cells at stages of gonadal ridge development in 46,XX and 46,XY individuals. Differential gene expression analysis revealed significantly upregulated genes. Within these, we identified *CITED2* as a gene containing a MYRF binding site. It has been shown that *Cited*2^−/−^ mice have gonadal defects in both testis and ovary differentiation, as well as defects in heart development and establishment of the left–right axis. This makes *MYRF* a potential candidate as an early regulator of gonadal and heart development via upregulation of the transcriptional cofactor *CITED2*.

## 1. Introduction

In 2009, the *MYRF* gene (*Myelin Regulatory Factor*, OMIM * 608329) was described as a critical transcriptional regulator required for the myelination of the central nervous system (CNS) [1]. Subsequently, studies characterized the function of *MYRF* as a membrane-associated transcription factor for oligodendrocyte differentiation, stimulating myelin gene expression in the CNS [2,3]. Currently, it is known that *MYRF* is also expressed in other tissues in addition to the CNS, especially during the early development of the diaphragm, heart, eyes, lungs and genitourinary tract, which is consistent with the fact that variants in this gene have been associated with congenital diaphragmatic hernia [4], congenital heart diseases [5], nanophthalmos and other ocular defects [6], and more recently, with a new cardiac and urogenital syndrome (CUGS), as well as with disorders/differences of sex development (DSD) [7,8], a group of conditions in which development of chromosomal, gonadal or anatomic sex is atypical [9]. Sex determination in mammals is controlled by the interaction between many genes that drive a bistable embryonic gonadal development program. Testis and ovarian promoting pathways act as an antagonistic network system with a remarkable plasticity [10]. In the 46,XY gonad, the presence of SRY activates the *SOX9/FGF9* pathway, leading to testis development, Sertoli and Leydig cell formation and androgen production, resulting to internal and external virilization. In contrast, the absence of *SRY* in the 46,XX gonad will upregulate the *RSPO1/WNT4/b-catenin* pathway, repressing the *SOX9/FGF9* pathway to promote ovarian development, with granulosa and theca cells. In humans, a disruption in either pathway can result in gonadal dysgenesis, leading to a range of genital phenotypes between both sexes [11].

*MYRF* (previously known as *C11orf9* [12]), is located at 11q12.2 and expands around 35 kb of genomic DNA divided into 27 exons. It encodes for a 1151 amino acid protein, characterized by a proline-rich region containing a nuclear localization signal (NLS), a DNA-binding domain (DBD), an intramolecular chaperone auto-processed domain (ICA) (also known as Peptidase S74 domain), a transmembrane domain (TM) and two myelin gene regulatory factor C-terminal domains (MRF C1 and MRF C2) [3,12]. An intramolecular chaperone from the ICA domain helps *MYRF* to form a homo-trimer in the endoplasmic reticulum, which is essential for its biological process, where it carries out the auto-cleavage of *MYRF*, allowing it to function as a transcription factor in the nucleus [13].

Here, we report a novel, heterozygous de novo p.Q838* variant in *MYRF* in a patient with 46,XY partial gonadal dysgenesis (PGD), Scimitar syndrome and severe hyperopia, identified by whole genome analysis. Haploinsufficiency of *MYRF* has been identified as a cause of DSD in 46,XX and 46,XY individuals [7]; however, there is limited knowledge about the function of this gene and its influence on the molecular pathways in sex development. To elucidate the influence of *MYRF* expression on the developing gonads, we use previously published single cell RNA-seq data of human testes and ovaries. *CITED2* was found upregulated in *MYRF*-expressing cells and is possibly a downstream target of *MYRF*. We highlight the important role *MYRF* plays in early developmental stages of the undifferentiated gonad and how it can affect gonadal development of 46,XY and 46,XX individuals, as well as heart morphogenesis and establishment of the left–right axis.

## 2. Materials and Methods

### 2.1. Patient Description

The child was prenatally suspected of having a congenital heart defect with questionable situs inversus and anomalous pulmonary vein confluence. The birth was by caesarean section at gestational week 38 + 5. Genital status was not questioned at that time and a female sex was assigned. Scimitar syndrome was diagnosed with dextrocardia and an abnormal confluence of a right-sided pulmonary vein in the inferior vena cava. The right lung showed a rudimentary upper lobe bronchus that narrowed after 1 cm. Only one dorsal relatively small right upper lobe segment was present. The right lower lobe was pushed dorsally by the heart and was considerably compressed and partly atelectatic. Moreover, also belonging to the right lung, an overinflating lung segment was seen ventrobasally to the left of the heart. The well ventilated and fully expanded left lung was considerably larger than the right one. In addition, aside of the spleen, two small accessory spleens were seen. At the age of 6 months, the anomalous pulmonary veins became attached to the left atrium. A follow-up at the age of one year showed rarefaction in the right lower lobe of the lung.

At the age of 8 years, clitoris hypertrophy without signs of puberty became apparent and the child was seen by a paediatric endocrinologist. Apparently, both gonadotrophins and testosterone were elevated. She presented to our hospital endocrine unit at the age of 10 years for further assessment. At this time, she felt healthy and there were no imminent pulmonary or cardiovascular problems. She had no acne, no hirsutism, no breast development and was of normal height and weight. She had severe hyperopia with 9 dpt, corrected with glasses. Her clitoris was 3.5 cm in length and the labia majora were rugated. LH was 5.7 IU/L (within the adult range), FSH 69.9 IU/L (above adult range), testosterone 0.51 µg/L (elevated for the female reference range, within the reference range of Tanner 2 pubertal development for boys). Additionally, 17-beta estradiol was below detection and inhibin B, at 9 pg/mL, was below the reference range for boys. On ultrasound, she had a small tubular structure behind the bladder, which was interpreted as a small rudimentary uterus. This was confirmed by MRI; gonads could not be visualised at that time. Urine steroid metabolome analysis revealed no signs of congenital adrenal hypoplasia. Subsequent karyotype analysis was 46,XY (ishYP11.3, SRY positive). Taken together, the findings were consistent with a 46,XY PGD. Puberty was stopped by a GnRH analogue. After gender assessment by a psychologist, puberty was induced at an appropriate age by estrogens/progestins. During follow-up, small gonads could be visualized by ultrasound or MRI in the inguinal canal and were examined every 12 months. Prophylactic gonadectomy was discussed repeatedly and a shared decision was made to delay the procedure until after her 18th birthday. Dextrocardia and hyperopia in conjunction with gonadal dysgenesis suggested a syndromic form of DSD. Array-CGH using a 180 k Microarray (Agilent) revealed an unsuspicious 46,XY karyotype without clinical relevant deletions or duplications (arr [hg19] 1–22)x2,(XY)x1). To elucidate the cause of this form of DSD, a whole genome sequencing analysis was initiated.

### 2.2. Whole Genome Sequencing (WGS)

WGS was performed to identify putative pathogenic variants, indels and structural variations. Sequencing libraries were constructed from 1.0 µg DNA per sample using the Truseq Nano DNA HT Sample Preparation Kit (Illumina, San Diego, CA, USA), following the recommendations of the manufacturer. Genomic DNA was randomly fragmented to a size of approximately 350 bp by Covaris cracker (Covaris, Woburn, MA, USA). DNA fragments were blunted, A-tailed and ligated with the full-length adapter for Illumina sequencing with further PCR amplification. Libraries were purified using AMPure XP (Beckman Coulter, Pasadena, CA, USA), analysed for size distribution on an Agilent 2100 Bioanalyser (Agilent Technologies, Santa Clara, CA, USA) and quantified by qPCR. Paired end sequencing of libraries was performed on Illumina HiSeq platforms (Illumina). Per sample, more than 90 GB of raw data were obtained, resulting in an average genomic read depth of 30×.

### 2.3. Bioinformatics

Sequencing reads were mapped to human reference genome version GRCh38 using the Burrows–Wheeler Aligner (BWA-MEM, v0.7.17) [14], and resulting mapping files were processed following GATK (Genome Analysis Toolkit) best practices for germline variant calling. Briefly, we screened for duplicated reads by applying *Mark Duplicates* from Picard tools (v.2.26.1, http://broadinstitute.github.io/picard/, accessed on 3 September 2021); split-reads and discordant paired-end alignments were extracted using *SAM tools* v0.1.18 [14] as implemented in (GATK, v.4.2.2.0) [15] with standard parameters. Single nucleotide variants (SNVs) and short insertions/deletions (InDels) were called using *Deep Variant* (v1.2.0). Detection of structural variants (SV) was performed using DELLY [16]. Variants were annotated using Ensembl Variant Effect Predictor—VEP (v103) (EMBL-EBI, Cambridge, UK). All chromosomal positions in the paper are according to GRCh38.

### 2.4. Single Cell RNA Analyses

We analysed publicly available single-cell RNA from testes and ovaries, which totalled 20 samples from testes of individuals at different stages, from embryo to adult men, and 4 samples of ovaries, from embryos and foetus [17,18,19,20] (for more detail, see the *Data Availability* section below and Supplementary Table S1). The scRNA sequences of these samples were obtained through the 10x Genomics sequencing platform. We pre-processed the raw sequences using fastp (v0.22.0) [21], which were later aligned and annotated to the human reference cDNA GRCh38 with Kallisto (v0.46.1) and BUStools (v0.39.3) [22]. This resulted in matrixes of UMI counts for each sample. The downstream filtering and analyses were performed with the R package Seurat v4.1.1 [23]. In brief, we retained genes expressed in at least three cells, and we kept cells if they had less than 25% reads mapping to the mitochondrial genome, if they had between 500 and 9000 genes, and if they had less than 40,000 mRNA molecules detected. We normalized the gene counts in each cell by the total cell expression, with the *Normalize Data* function, using log10-transformation and a scale factor of 10,000. Integration of the samples was performed for testes and ovaries datasets separately, using *FindIntegration Anchors* and *Integrate Data* functions. Afterward, the cells were clustered by a shared nearest neighbour (SNN) modularity optimization and visualized in a two-dimensional space based on uniform manifold approximation and projection (UMAP). We classified the cell populations using the same marker genes as in the original publications [17,18,19,20] and other published literature [24,25].

To investigate which genes could have their expression affected by *MYRF*, we first subset the cell cluster of the coelomic epithelium (CE), the cell population that expressed this gene the highest. Later, we performed a differential expression analysis between the cells that express *MYRF* against those that do not express it, using the *FindMarkers* function with default parameters.

## 3. Results

**Figure 1:**
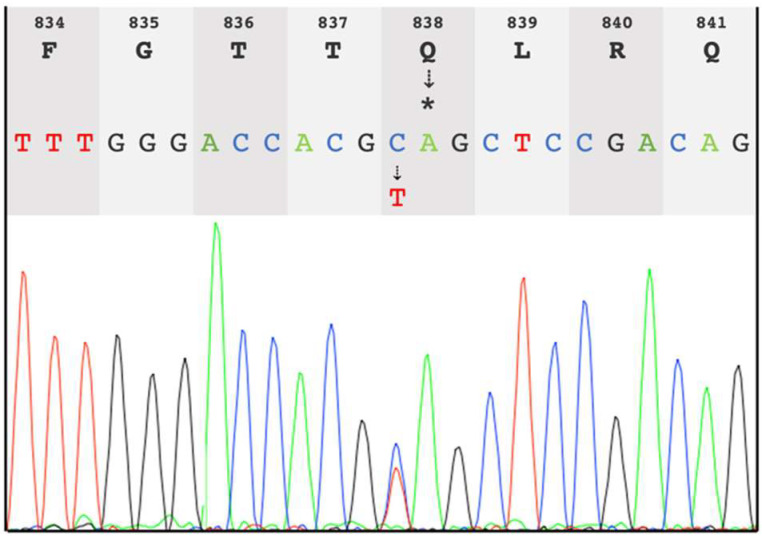
Sanger chromatogram showing the de novo heterozygous stop-gain variant in *MYRF* (c.2512C>T; p.Q838*) in the study patient.

We performed Trio-WGS (mother, father and affected child) and focused our analyses on protein-coding regions. SNVs and Indels were filtered for rare variants (frequency in 1 k project or gnomAD below 0.1%). A heterozygous de novo stop-gain variant in exon 20 of the myelin regulatory factor (*MYRF*) was detected (NM_001127392:c.2512C>T; p.Q838*) and confirmed by Sanger sequencing (Figure 1). According to the ACMG guidelines [26], this variant was considered pathogenic as it fulfils two criteria of the evidence of pathogenicity: PSV1 (classified as “null variant” since it is a nonsense variant) and PS1 (de novo variant confirmed by the absence in both parents). De novo variants in *MYRF* have been recently detected in patients with CUGS [27]. No further variants in genes associated with gonadal dysgenesis have been detected.

Since variants in *MYRF* have been recently detected not only in 46,XY, but also 46,XX gonadal dysgenesis [7], we hypothesized that heterozygous inactivating variants may affect very early development of the bipotential gonad. Therefore, we reanalysed publicly available single cell RNA sequencing data (scRNA-seq) of different stages of human gonadal development to study the impact that *MYRF* could have in the early development of the gonads.

**Figure 2:**
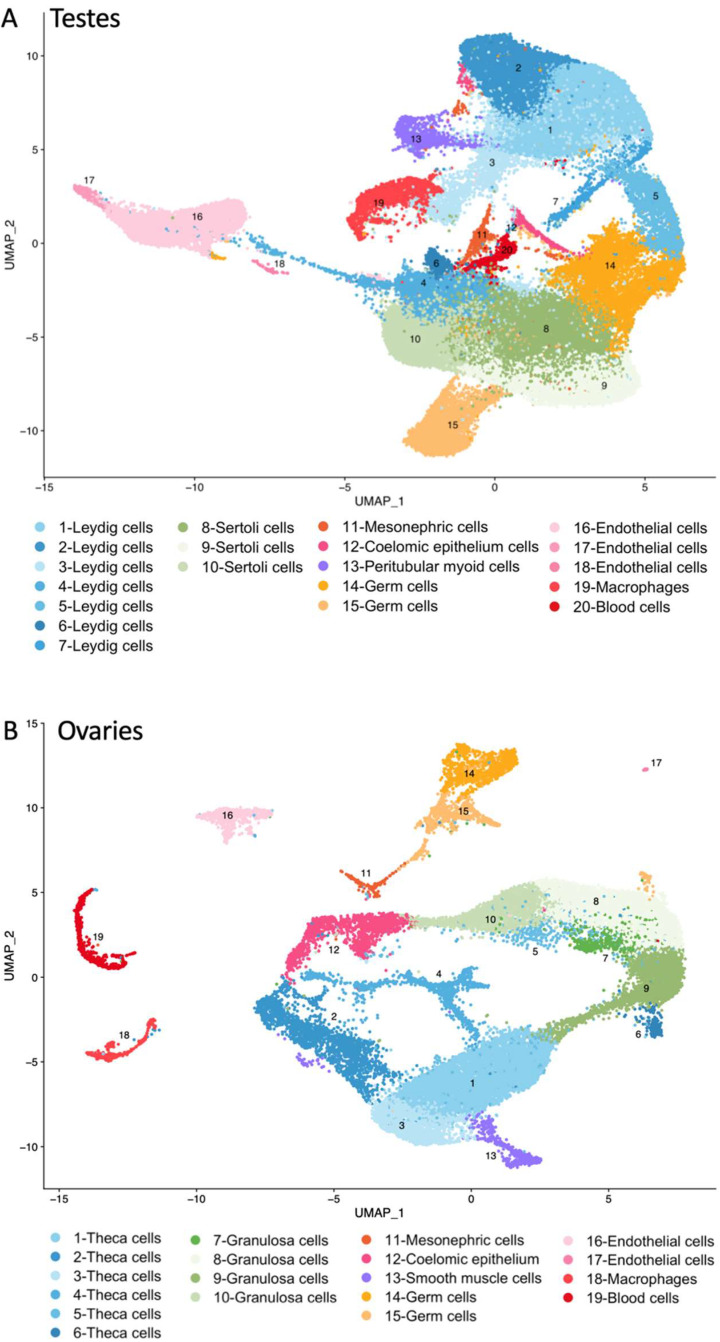
UMAP plots of cell clusters in human (**A**) testes from embryonic stage to adults; and (**B**) ovaries from embryonic and foetal stages identified by single cell RNA sequencing. UMAP: uniform manifold approximation and projection.

According to their stage or age, the testis samples were classified as embryonic, foetal, mini puberty, peri-puberty, pre-puberty and adults, and the ovary samples were classified as embryonic or foetal (Supplementary Table S1). The reanalysis of these datasets yielded ~111,000 cells from testes and ~28,000 cells from the ovaries, which clustered in 20 and 19 cell populations, respectively (Figure 2), which were identified according to their distinctive gene expression (Supplementary Figures S1 and S2). In testes, clusters 1–7 were annotated as Leydig cells, clusters 8–10 as Sertoli cells. Cluster 11 was classified as mesonephric cells; however, it was also composed of cells with gene expression patterns of Leydig and Sertoli cells and was present mainly in the embryos and foetus. Cluster 12 was identified as coelomic epithelium cells and was also present mainly in embryos and foetus. Cluster 14–15 belonged to germ cells. The remaining clusters were classified as peritubular myoid, endothelial, macrophages and blood cells (Figure 2A). In ovaries, theca cells were found in cluster 1–6, granulosa cells in cluster 7–10, mesonephric cells in cluster 11 and coelomic epithelium cells in cluster 12. Germ cells were present in cluster 14–15 and the remaining cells were classified as smooth muscle, endothelial, macrophages and blood cells (Figure 2B).

**Figure 3:**
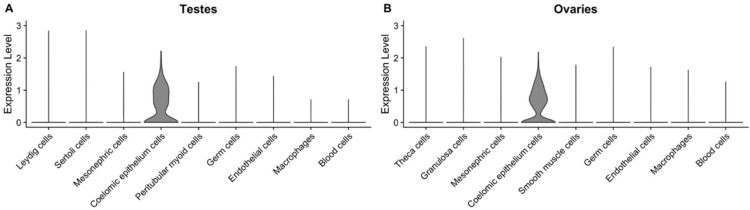
Expression levels of *MYRF* in testes (**A**) and ovaries (**B**) at embryonic and fetal stages, showing that it is highly expressed in coelomic epithelium cells.

We observed that, in both testes and ovaries, *MYRF* is highly expressed in a subset of cells from the coelomic epithelium (CE) at embryonic and foetal stages (Figure 3), and that its expression is lowered after birth (Supplementary Figure S3). We aimed to search for genes that might be directly or indirectly regulated by this transcription factor. Therefore, we divided the cluster of CE cells into cells that express *MYRF* and those that do not, and investigated them for differentially expressed (DE) genes. We found 62 DE genes in the CE cells of testis and 13 genes in the CE of ovaries. Within this set of DE genes, approximal half of them (26 of the testis and 7 of the ovary genes) have *MYRF* binding sites according to the *GeneHancer* database [28] (Table 1 and Table 2). One of these DE genes, the *CBP/p300-interacting transactivator with Glu/Asp-rich carboxy-terminal domain 2* (*CITED2*), is of particular interest, since it is a transcriptional cofactor that acts in the early stages of XX and XY gonadal development [29,30], and is involved in heart morphogenesis and establishment of the left–right axis [31].

## 4. Discussion

The *MYRF* gene encodes a membrane-associated transcription factor, which has been recently identified as candidate of a new syndrome of cardiac and urogenital anomalies (CUGS) [27,32]. Shortly afterwards, further de novo variants in *MYRF* have been identified as the genetic cause of congenital diaphragmatic hernia (CDH) in patients that also presented congenital heart disease (CHD); three of the patients also had varying genital anomalies ranging from female external genitalia with a blind ending vagina to ambiguous genitalia and/or undescended testes, extending the spectrum of CUGS [4].

Additionally, variants in *MYRF* have been found in patients with eye conditions. For instance, Garnai and colleagues [6] identified a frameshift variant (c.769dupC, p.S264Qfs*74) in an eight year old male subject with nanophtahalmos, an extreme form of hyperopia or farsightedness, who also presented cardiac and urogenital malformations, such as a mitral valve prolapse, a micropenis and unilateral cryptorchidism. In the same way, Xiao and colleagues [33] found a de novo frameshift variant (c.3274_3275del, p.L1093Pfs*22) in a six year old boy with high hyperopia and a splice-site variant (c.3194 + 2T>C) in a 40-year-old woman with high hyperopia that developed angle-closure glaucoma of both eyes. Familial cases of nanophthalmos due to disrupting variants of *MYRF* have also been identified. Garnai and colleagues also studied a large family with inherited nanophthalmos, and found a disrupting splice site variant (c.3376-1G>A, p.G1126Vfs*31), leading to a frameshift in the last exon. Four members of this family also had dextrocardia, and one of them also had a right hypoplastic lung and pulmonary artery stenosis. Shortly after, Xiao and colleagues [33], investigating another large family with inherited nanophthalmos, revealed an additional truncating mutation in *MYRF* (c.3377delG, p.G1126Vfs*31). Although the variants in the two large families identified by Garnai et al. [6] and Xiao et al. [33] were different, the effect on the protein was the same. Siggs and colleagues [34] also identified a large family with inherited autosomal nanophthalmos associated with a C-terminal frameshift variant in *MYRF* (c.3361delC, p.R1121Gfs36*) introducing a premature terminal codon in the penultimate exon. These familial cases of nanophthalmos are mostly non-syndromic, leading to the speculation that the ocular tissue is more sensitive to haploinsufficiency than the cardiac and urogenital tissues [6]. On the other hand, little is known so far about the function of the C-terminal part of MYRF located in the ER membrane.

In addition to the association between MYRF variants with congenital heart defects and nanophthalmos, Hamanaka et al. described *MYRF* as a new candidate gene for DSD [7], after a case-control study comparing gene-based burdens of rare damaging variants between both groups. The authors described four new *MYRF* variants in five individuals from four families. From those, three patients were 46,XY and two were 46,XX. All of them presented different phenotypes of DSD; however, only one had a diaphragmatic hernia, and neither heart defects nor nanophthalmos were associated in those patients. More recently, Globa et al. [8] reported two other cases of *MYRF* heterozygous variants as a cause of DSD. Both individuals were 46,XY; the first one registered as a female with ambiguous genitalia, later raised as a boy, and the second one was a girl with primary amenorrhea, a lack of secondary sexual characteristics and high-grade hypermetropia. According to the authors, the *MYRF* loss-of-function variant described in this patient was the first case combining gonadal dysgenesis and nanophthalmos phenotypes. Here, we present the case of a 46,XY patient with PGD, Scimitar syndrome, pulmonary vein malformation and severe hyperopia, who, to the best of our knowledge, is the first case involving eye, heart and genital defects due to a *MYRF* disruptive variant.

Although the function of this gene in sex development pathways is still unclear, the association of heterozygous de novo variants with gonadal dysgenesis in both 46,XY and 46,XX individuals points to an important function in early gonadal development [4,7]. Based on our bioinformatics analyses utilizing scRNA-seq, we bring robust evidence that *MYRF* is expressed mainly in cells of the coelomic epithelium during early development of the bipotential gonads. Similar results were reported by Hamanaka et al. in 2019, using a different scRNA data set, thus corroborating our results. The coelomic epithelium constitutes the first layer of cells in the urogenital ridge at the fourth week post fertilization (wpf), and during the fifth wpf they proliferate, forming a thickened multi-layered cluster of cells that differentiates into the primordial gonad or gonadal ridge. Some of the CE cells transform into NR5A1-positive gonadal precursors that later differentiate into Sertoli and granulosa cells, as well as into steroidogenic cells [35]. At the same time, the primordial germ cells migrate from the hindgut into the NR5A1-positive gonadal precursor cells [36,37]. CE cells have an important role in the early development of the bipotential gonads. Therefore, disrupting variants in the *MYRF* gene, which is highly expressed in this cell population, could have an impact in the normal development of the gonads.

Of the differentially expressed genes with a binding site for *MYRF*, *CITED2* (OMIM * 602937) caught our special attention. *CITED2* is involved in early gonadal development and sex determination. In mice, *Cited*2 is expressed in the urogenital ridge and cooperates with *Wt1* to stimulate the transcription of *Nr5A1* [29,38]. In addition, a physical interaction of CITED2 and WT1 was shown by immunoprecipitation (IP), as well as GST pulldown, revealing an interaction with both WT1 isoforms (^+/−^KTS) [38]. Buaas et al. had shown that CITED2 functions to ensure high expression levels from *Nr5a1 and Sry*, providing a proper activation of the male pathway during sex development, and providing evidence that *Sry* is a downstream target of the CITED2/WT1/NR5A1 regulatory pathway [29]. Moreover, reduced gene dosage of *Wt1* or *Nr5a1* in *Cited2* mutant gonads leads to partial sex reversal and can undergo complete XY sex reversal in the context of a hypomorphic *Sry* allele [29]. A transcriptional delay of male development in *Cited2*^−/−^ mice was reported by Combes et al. [30], due to a reduction of *Nr5a1* levels at early gonadal development. Furthermore, XX *Cited2*^−/^^−^ gonads showed defects in ovary differentiation with ectopic cell migration, transient upregulation of testis promoting factor *Fgf9* and delay of ovary promoting genes *Wnt4* and *Foxl2*, suggesting *Nr5a1* as an ovarian promoting gene. Human variants in *CITED2*, such as in *NR5A1*, are associated with premature ovarian failure (POF) [39,40]. Our finding that, in humans, *CITED2* is found upregulated in *MYRF* positive cells of the coelomic epithelium of the ovaries and contains a *MYRF* binding site makes *MYRF* a potential candidate as a new upstream regulator of early gonadal development. Therefore, haploinsufficiency of this gene may lead to different forms of gonadal dysgenesis in 46,XX and 46,XY individuals, and *CITED2* might be an important downstream target of *MYRF*.

Interestingly, *CITED2* is also required for heart morphogenesis, as well as establishment of the left–right axis in humans and mice [31,41,42]. *Cited2*^−/−^ mice die during gestation with diverse cardiovascular malformations, such as atrial and ventricular septal defects (ASD/VSD), aberrant aortic arches, and common arterial trunk and left–right patterning defects [31,42], a phenotype very similar to that of patients with heterozygous de novo variants in *MYRF*. In humans, variants in *CITED2* were identified in patients with congenital heart defects (CHD) such as sinus venosus atrial septal defects, abnormal pulmonary venous return to the right atria, Tetralogy of Fallot, dextrocardia and other ventricular septal defects [41,43], also seen in patients with *MYRF* variants [44]. More importantly, several of these variants are localized in the promoter region of *CITED2* and have been proven to alter *CITED2* transcriptional activity, decreasing their expression [41,45]. Therefore, it is possible that haploinsufficiency of transcription factors of *CITED2*, such as *MYRF*, can influence its expression levels and lead to heart defects.

It is also important to highlight that according to an in silico prediction of the genome aggregation database (gnomAD v2.1.1) *MYRF* is a constraint gene and the value of pLI (probability of being loss-of-function intolerant) is 1, supported by a very low LOEUF (loss-of-function observed/expected upper bound fraction) value of 0.117, which means that this is a haploinsufficient gene, where loss of a single copy is not tolerated [46]. This is corroborated by the finding that, such as in our case, de novo variants are associated with most of the syndromic forms. In contrast, familiar cases of nanophthalmos are mostly associated with variants in the last exon, and other features of the CUGS are only seen in some individuals [6,33,34]. In the same way, no loss-of-function variants have been observed so far in *CITED2* in gnomAD, making *CITED2* a potential candidate of a downstream factor of *MYRF* whose reduced expression may induce the observed phenotypes in humans. Haploinsufficiency is frequently observed in cases of DSD produced by heterozygous variants or copy number variations in transcriptions factors such as *NR5A1*, *SOX9*, *NR0B1* or *WT1*, demonstrating that sex determination is highly sensitive to gene dosage [47,48,49]. Therefore, phenotypic variability of *MYRF* haploinsufficiency on gonadal development was expected since it acts in the same pathway. Nevertheless, further epigenetic and transcriptome studies in the respective gonadal and/or heart tissues will be necessary to elucidate the pleiotropic developmental effects of *MYRF* variants in the context of gene regulation and protein interaction.

## 5. Conclusions

Our data give evidence that heterozygous inactivating variants in *MYRF* may influence the upregulation of *CITED2* and thereby impact the *NR5A1* pathway of sex development very early in the gonadal ridge of both sexes, which might make *MYRF* haploinsufficiency a feasible cofactor disease due to missing upregulation of *CITED2*. Further investigations are needed to elucidate the interaction between *MYRF* and *CITED2*. In addition, a missing upregulation of *CITED2* by *MYRF* in heart development might also be the cause for abnormal pulmonary venous return to the right atria, atrial septal defect (ASD) or tetralogy of Fallot (ToF), as well as a left–right patterning defect that is observed in humans with heterozygous *CITED2* variants and *Cited2*^−/−^ mice [31,41]. Heterozygous variants in *MYRF* are also associated with nanophthalmos. To our knowledge, we present the first case with a combination of three *MYRF* associated phenotypic features: heart defects (CHD, dextrocardia, anomalous pulmonary venous return), 46,XY PGD and severe hyperopia. A reason for this might be the very recent description of the CUGS, as well as the separate detection of C-terminal *MYRF* variants in familiar cases of nanophthalmos without further CUGS symptoms and the different time points of diagnosis. While cardiac failures are detected at birth, nanophthalmos might be only detected during the first years of childhood, and gonadal dysgenesis is often only detected at puberty, all by different specialists, making a common diagnosis often difficult.

## Figures and Tables

**Table 1:** List of genes differentially expressed between the cells that express *MYRF* and those that do not, in the coelomic epithelium cluster of ovaries.

Female Samples									
DE Genes	Binding Site for *MYRF*	pct. *MYRF* (+)	pct. *MYRF* (−)	*p*-Value adj	DE Genes	Binding Site for *MYRF*	pct. *MYRF* (+)	pct. *MYRF* (−)	*p*-Value adj
*MYRF*	+	1	0	1.3608 × 10^−261^	*ZFP36*	+	0.543	0.398	3.03465 × 10^−5^
*NR4A1*	−	0.444	0.249	1.63171 × 10^−10^	*IER2*	+	0.81	0.691	0.000188878
*SOCS3*	−	0.443	0.239	2.54304 × 10^−10^	*CITED2*	+	0.672	0.474	0.000278465
*CRIP1*	−	0.509	0.322	5.59507 × 10^−8^	*CALB2*	−	0.439	0.292	0.000329296
*FOSB*	+	0.568	0.383	1.88434 × 10^−7^	*JUNB*	+	0.642	0.513	0.001629543
*EGR1*	+	0.631	0.464	1.82088 × 10^−6^	*FOS*	−	0.775	0.693	0.006444703
*DUSP1*	+	0.644	0.459	3.9062 × 10^−6^					

DE: differential expression analyses. *MYRF* (+): cells from coelomic epithelium that express at least one transcript of *MYRF. MYRF* (−): cells from coelomic epithelium that do not express any transcript of *MYRF.* Pct: percentage of cells where the gene is detected in *MYRF* (+) or (−) subsets.

**Table 2:** List of genes differentially expressed between the cells that express *MYRF* and those that do not, in the coelomic epithelium cluster of testes.

Male Samples									
DE Genes	Binding Site for *MYRF*	pct. *MYRF* (+)	pct. *MYRF* (−)	*p*-Value adj	DE Genes	Binding Site for *MYRF*	pct. *MYRF* (+)	pct. *MYRF* (−)	*p*-Value adj
*MYRF*	+	1	0	1.18 × 10^−81^	*TNNI1*	−	0.764	0.45	0.001116
*H2AFX*	+	0.651	0.282	6.08 × 10^−8^	*TMEM88*	−	0.455	0.187	0.001454
*C21orf58*	−	0.371	0.1	1.29 × 10^−6^	*GGH*	−	0.48	0.201	0.001921
*HILPDA*	−	0.535	0.201	2.98 × 10^−6^	*UBE2T*	−	0.455	0.211	0.001941
*DSG2*	+	0.549	0.211	3.24 × 10^−6^	*MKI67*	−	0.404	0.163	0.002216
*DNMT1*	+	0.6	0.263	3.39 × 10^−6^	*ZWINT*	−	0.484	0.22	0.002686
*ROBO1*	−	0.484	0.172	8.73 × 10^−6^	*CDK1*	−	0.418	0.172	0.002747
*AURKB*	+	0.458	0.163	2.32 × 10^−5^	*IDH2*	+	0.698	0.407	0.003044
*SGO1*	−	0.371	0.11	3.15 × 10^−5^	*CDCA3*	−	0.313	0.1	0.004612
*GMNN*	+	0.524	0.225	3.97 × 10^−5^	*PABPN1*	+	0.76	0.488	0.004802
*NOVA1*	−	0.716	0.383	4.61 × 10^−5^	*MTRNR2L10*	NA	0.825	0.526	0.006358
*STX10*	+	0.585	0.258	7.52 × 10^−5^	*PCNA*	+	0.665	0.373	0.006529
*UBE2S*	+	0.625	0.335	9.06 × 10^−5^	*TYRO3*	+	0.633	0.325	0.008898
*CMSS1*	−	0.622	0.297	0.000107	*ECI1*	+	0.596	0.306	0.009338
*FEN1*	−	0.447	0.172	0.000119	*TK1*	+	0.564	0.325	0.009615
*SCARB2*	+	0.629	0.306	0.000145	*EIF3E*	+	0.982	0.967	0.010135
*RPSAP52*	−	0.444	0.172	0.000187	*LAPTM4B*	−	0.895	0.67	0.011395
*HNRNPAB*	+	0.716	0.388	0.000202	*CKS2*	+	0.695	0.426	0.014009
*YLPM1*	−	0.407	0.148	0.000275	*RABL6*	−	0.651	0.368	0.01407
*LIG1*	+	0.349	0.11	0.000322	*CENPF*	−	0.524	0.282	0.015355
*SMC1A*	−	0.455	0.182	0.000419	*AKR1B1*	+	0.876	0.617	0.020538
*SMC2*	−	0.498	0.225	0.000429	*BIRC5*	+	0.545	0.311	0.021154
*H2AFZ*	−	0.942	0.823	0.00049	*AURKA*	+	0.255	0.077	0.021308
*CENPM*	−	0.44	0.177	0.000496	*CBX5*	−	0.931	0.732	0.022631
*CENPV*	−	0.647	0.368	0.000586	*SMARCC1*	+	0.771	0.464	0.022672
*USP10*	−	0.531	0.244	0.000751	*UBE2C*	+	0.542	0.297	0.023105
*CDH3*	−	0.531	0.249	0.000837	*ZDHHC8P1*	−	0.742	0.464	0.027005
*ASF1B*	+	0.302	0.086	0.000849	*MAG*	−	0.495	0.249	0.031231
*HNRNPD*	+	0.884	0.718	0.00087	*SOX11*	−	0.64	0.383	0.031795
*ITGA3*	−	0.596	0.292	0.000892	*TPX2*	+	0.375	0.153	0.035526
*TMEM106C*	−	0.68	0.411	0.000936	*H2AFV*	−	0.858	0.622	0.038892
*FBXO5*	−	0.367	0.129	0.000963					

DE: differential expression analyses. *MYRF* (+): cells from coelomic epithelium that express at least one transcript of *MYRF. MYRF* (−): cells from coelomic epithelium that do not express any transcript of *MYRF.* Pct: percentage of cells where the gene is detected in *MYRF* (+) or (−) subsets.

## Data Availability

The data presented in this study were previously published and are openly available in NCBI’s Gene Expression Omnibus [50], and are accessible through GEO Series accession number GSE143380 [20], GSE143356 [20], GSE124263 [17], GSE161617 [19] and GSE134144 [18].

## Supplementary Materials

The following supporting information can be downloaded at: www.mdpi.com/article/10.3390/jcm11164858/s1, Figure S1: Heatmap of the mean expression value in each cell cluster of the marker genes used to identify the cell populations found in testes samples; Figure S2: Heatmap of the mean expression value in each cell cluster of the marker genes used to identify the cell populations found in ovaries samples; Figure S3: Violin plot showing *MYRF* gene expression values in each cell population. Each dot represents the normalized expression value of *MYRF* in a cell. Cell clusters with less than ten cells were discarded. Table S1: List of samples reanalysed in this study derived from previous publications and deposited in the Gene Expression Omnibus database (GEO).
