# Supplementary material for: MYRF: A New Regulator of Cardiac and Early Gonadal Development—Insights from Single Cell RNA Sequencing Analysis
Source: J Clin Med. 2022 Aug 18;11(16):4858. doi: 10.3390/jcm11164858 (PMC9409872; doi:10.3390/jcm11164858)
Supplement: Supplementary file 1 [file jcm-11-04858-s001.zip › jcm-1819531-supplementary.pdf]

Supplementary Materials

Article:

MYRF: A new regulator of cardiac and early gonadal development - Insights from single cell RNA sequencing analysis

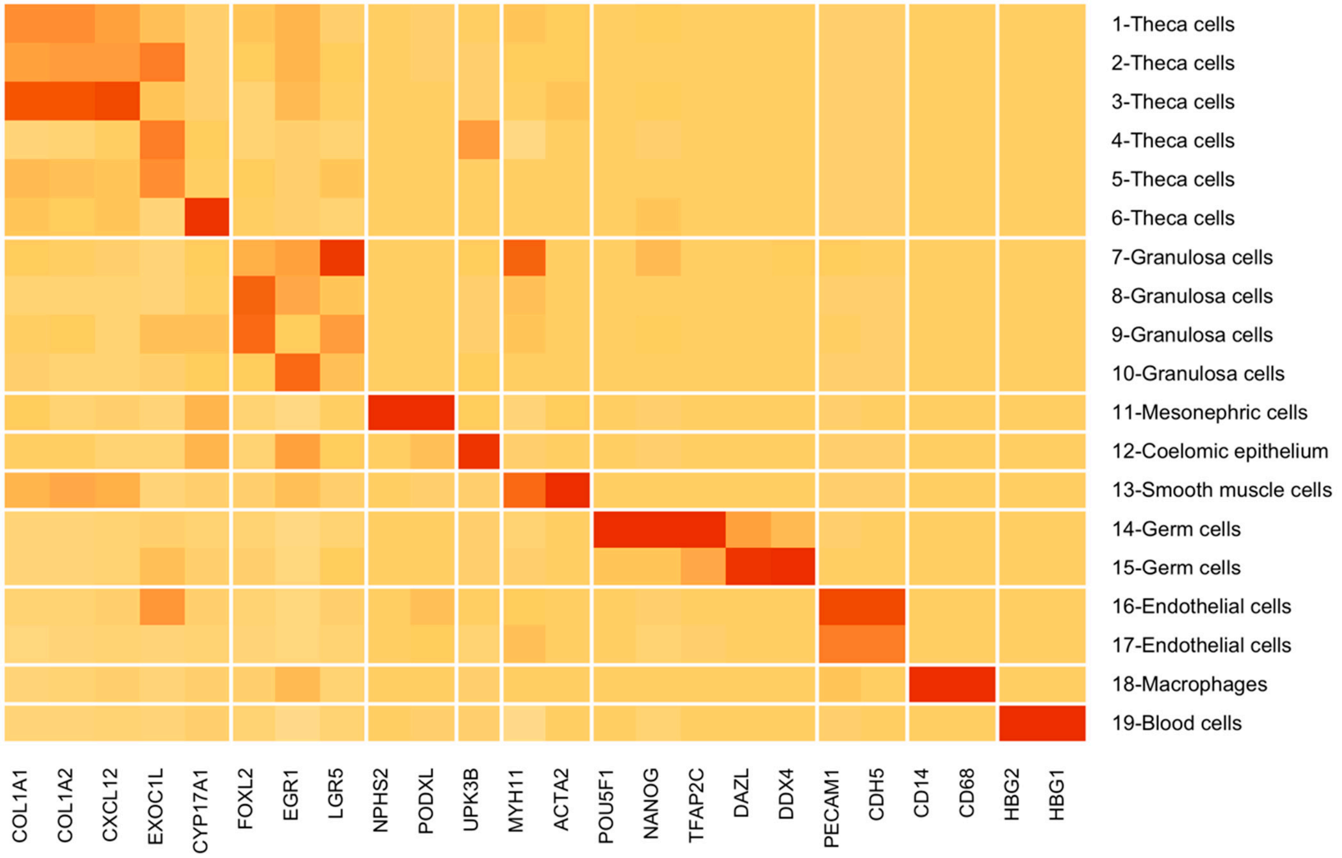

Figure S1. Heatmap of the mean expression value in each cell cluster of the marker genes used to identify the cell populations found in testes samples.

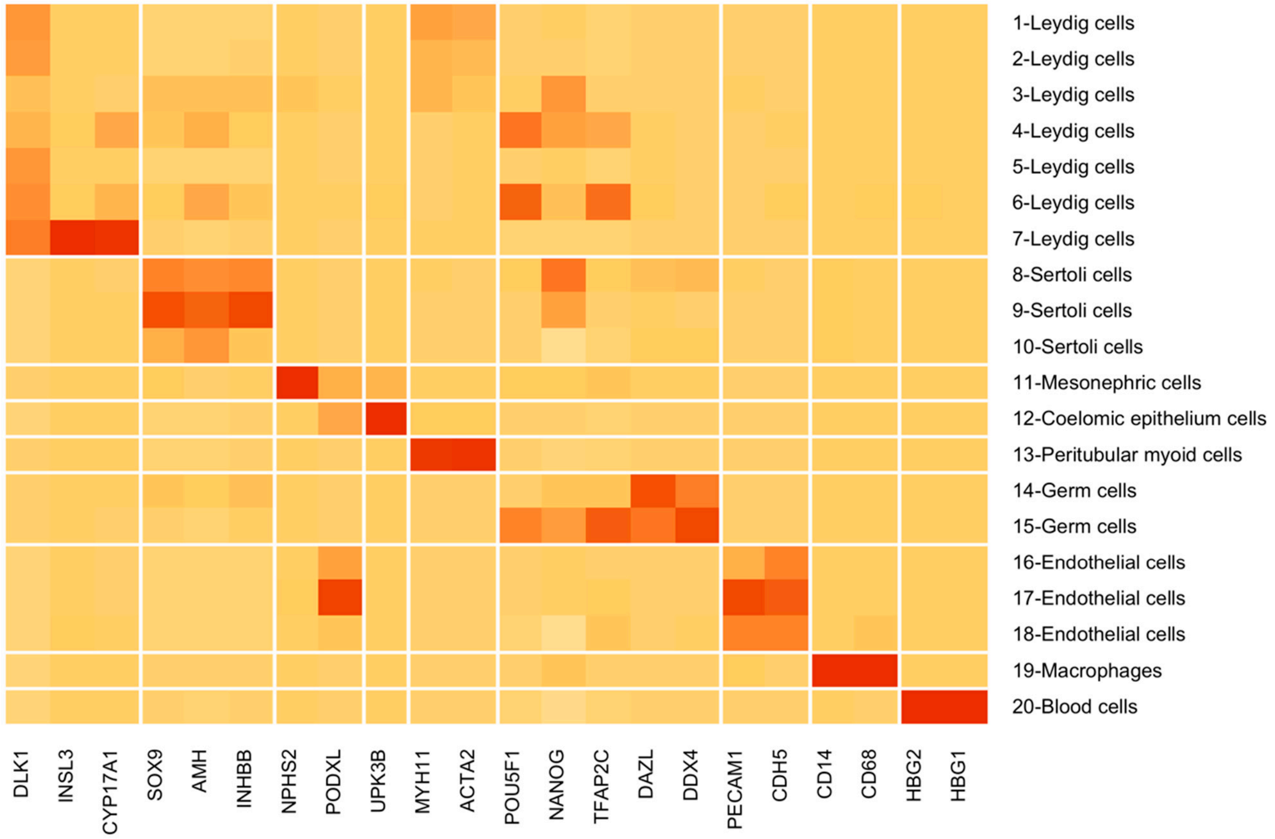

Figure S2. Heatmap of the mean expression value in each cell cluster of the marker genes used to identify the cell populations found in ovaries samples.

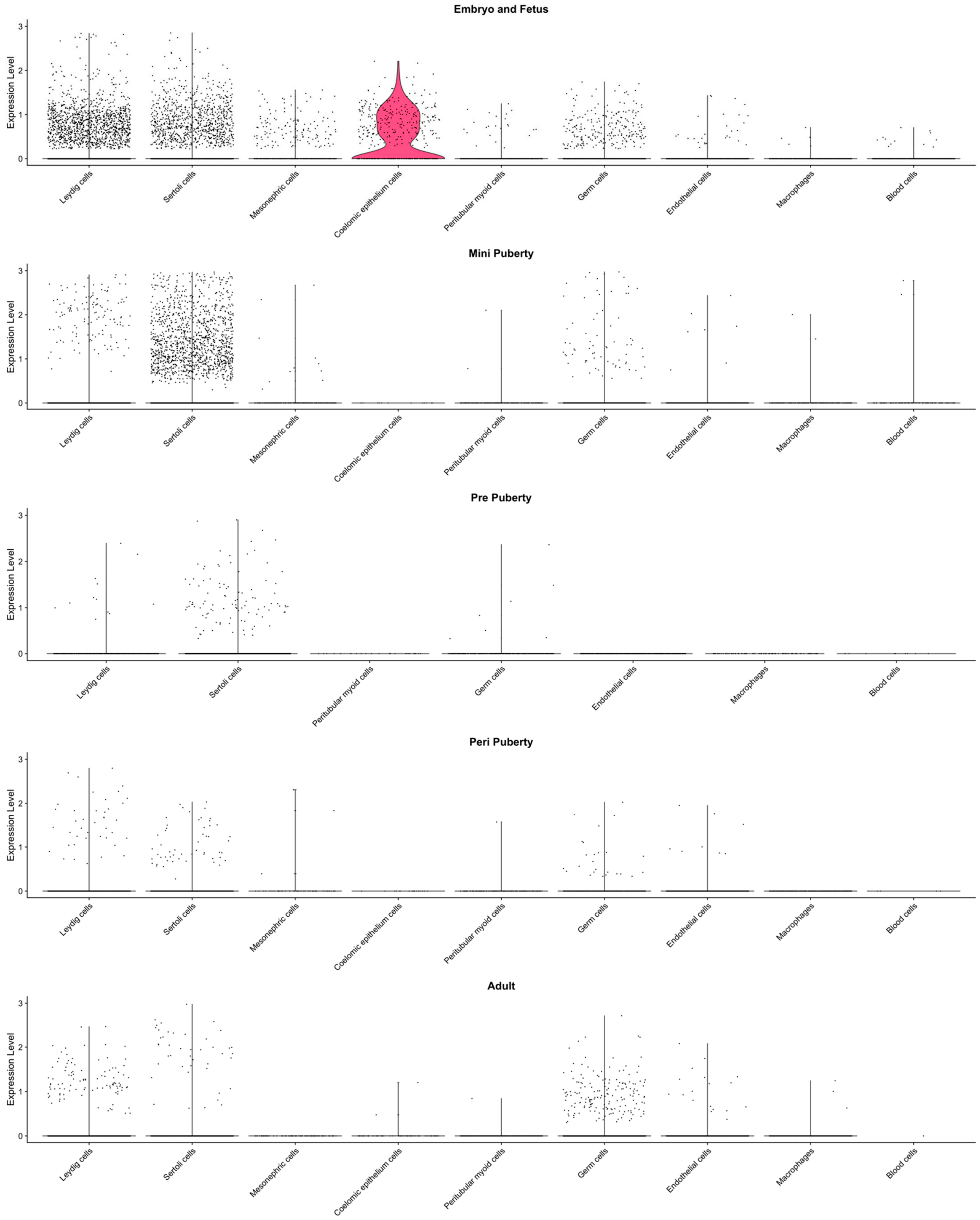

Figure S3. Violin plot showing *MYRF* gene expression values in each cell population. Each dot represents the normalized expression value of *MYRF* in a cell. Cell clusters with less than ten cells were discarded.

Table S1. List of samples reanalysed in this study derived from previous publications and deposited in the Gene Expression Omnibus database (GEO).

GEO Sample	GEO Series	Sample description	Cells origin	Group	Reference
GSM4257926	GSE143380	Week 7 post-fertilization	Ovary	Embryo	Chitiashvili, T., et al. (2020) [1] DOI: 10.1038/s41556-020-00607-4
GSM4257927	GSE143380	Week 9 post-fertilization	Ovary	Fetus	
GSM4257928	GSE143380	Week 10 post-fertilization	Ovary	Fetus	
GSM4257929	GSE143380	Week 13 post-fertilization	Ovary	Fetus	
GSM4257930	GSE143380	Week 16 post-fertilization	Ovary	Fetus	
GSM4257562	GSE143356	Week 6 post-fertilization	Testis	Embryo	
GSM4257563	GSE143356	Week 7 post-fertilization	Testis	Embryo	
GSM4257564	GSE143356	Week 8 post-fertilization	Testis	Embryo	
GSM4485991	GSE143356	Week 12 post-fertilization	Testis	Fetus	
GSM4257566	GSE143356	Week 15 post-fertilization	Testis	Fetus	
GSM4257567	GSE143356	Week 16 post-fertilization	Testis	Fetus	
GSM3526583	GSE124263	2 days old (ITGA6 enriched)	Testis	Mini puberty	Sohni, A., et al. (2019) [2] DOI: 10.1016/j.celrep.2019.01.045
GSM3526584	GSE124263	2 days old (unfractionated)	Testis	Mini puberty	
GSM3526585	GSE124263	7 days old (ITGA6 enriched)	Testis	Mini puberty	
GSM3526586	GSE124263	7 days old (unfractionated)	Testis	Mini puberty	
GSM4910868	GSE161617	5 months old (repetition 1)	Testis	Mini puberty	Guo, J., et al. (2021) [3] DOI: 10.1016/j.stem.2020.12.004
GSM4910869	GSE161617	5 months old (repetition 2)	Testis	Mini puberty	
GSM3937918	GSE134144	7 years olds (repetition 1)	Testis	Pre puberty	Guo, J., et al. (2020) [4] DOI:10.1016/j.stem.2019.12.005
GSM3937919	GSE134144	7 years olds (repetition 2)	Testis	Pre puberty	
GSM3937920	GSE134144	11 years olds (repetition 1)	Testis	Pre puberty	
GSM3937921	GSE134144	11 years olds (repetition 2)	Testis	Pre puberty	
GSM3937922	GSE134144	13 years olds (repetition 1)	Testis	Peri puberty	
GSM3937923	GSE134144	13 years olds (repetition 2)	Testis	Peri puberty	
GSM3937924	GSE134144	14 years olds (repetition 1)	Testis	Peri puberty	
GSM3937925	GSE134144	14 years olds (repetition 2)	Testis	Peri puberty	
GSM3526587	GSE124263	37 years olds (ITGA6 enriched)	Testis	Adult	Sohni, A., et al. (2019) [5] DOI: 10.1016/j.celrep.2019.01.045
GSM3526588	GSE124263	37 years olds (unfractionated)	Testis	Adult	
GSM3526589	GSE124263	42 years olds (ITGA6 enriched)	Testis	Adult	
GSM3526590	GSE124263	42 years olds (unfractionated)	Testis	Adult	

1. Chitiashvili, T.; Dror, I.; Kim, R.; Hsu, F.M.; Chaudhari, R.; Pandolfi, E.; Chen, D.; Liebscher, S.; Schenke-Layland, K.; Plath, K.; et al. Female human primordial germ cells display X-chromosome dosage compensation despite the ab-sence of X-inactivation. Nat. Cell Biol. 2020, 22, 1436–1446. https://doi.org/10.1038/s41556-020-00607-4.
2. Sohni, A.; Tan, K.; Song, H.W.; Burow, D.; de Rooij, D.G.; Laurent, L.; Hsieh, T.C.; Rabah, R.; Hammoud, S.S.; Vicini, E.; et al. The Neonatal and Adult Human Testis Defined at the Single-Cell Level. Cell Rep. 2019, 26, 1501–1517 e1504. https://doi.org/10.1016/j.celrep.2019.01.045.
3. Guo, J.; Sosa, E.; Chitiashvili, T.; Nie, X.; Rojas, E.J.; Oliver, E.; DonorConnect; Plath, K.; Hotaling, J.M.; Stukenborg, J.B.; et al. Single-cell analysis of the developing human testis reveals somatic niche cell specification and fetal germline stem cell establishment. Cell Stem Cell 2021, 28, 764–778 e764. https://doi.org/10.1016/j.stem.2020.12.004.
4. Guo, J.; Nie, X.; Giebler, M.; Mlcochova, H.; Wang, Y.; Grow, E.J.; DonorConnect; Kim, R.; Tharmalingam, M.; Matil-ionyte, G.; et al. The Dynamic Transcriptional Cell Atlas of Testis Development during Human Puberty. Cell Stem Cell 2020, 26, 262–276.e264. https://doi.org/10.1016/j.stem.2019.12.005.
5. Sohni, A.; Tan, K.; Song, H.W.; Burow, D.; de Rooij, D.G.; Laurent, L.; Hsieh, T.C.; Rabah, R.; Hammoud, S.S.; Vicini, E.; et al. The Neonatal and Adult Human Testis Defined at the Single-Cell Level. Cell Rep. 2019, 26, 1501–1517 e1504. https://doi.org/10.1016/j.celrep.2019.01.045.
